# Reduced culture temperature attenuates oxidative stress and inflammatory response facilitating expansion and differentiation of adipose-derived stem cells

**DOI:** 10.1186/s13287-019-1542-0

**Published:** 2020-01-23

**Authors:** Gal Tirza, Inna Solodeev, Meirav Sela, Ilanit Greenberg, Metsada Pasmanik-Chor, Eyal Gur, Nir Shani

**Affiliations:** 1 The Department of Plastic and Reconstructive Surgery, Tel Aviv Sourasky Medical Center, Tel Aviv, Israel; 2 The Bioinformatics Unit George S. Wise Faculty of Life Sciences, Tel Aviv University, Tel Aviv, Israel; 3 The Department of Plastic and Reconstructive Surgery, Tel Aviv Sourasky Medical Center, affiliated with the Sackler Faculty of Medicine, Tel Aviv University, Weizmann 6, Tel Aviv, Israel

## Abstract

**Background:**

Adipose-derived stem cell (ASC) expansion under atmospheric oxygen levels (21%) was previously shown to cause increased reactive oxygen species (ROS) accumulation and genetic instability compared to cells cultured under physiological oxygen levels (2–8%). However, since culture under physiological oxygen levels is costly and complicated, a simpler method to reduce ROS accumulation is desirable. The current study aimed to determine whether lower culture temperature can reduce ROS production in ASCs without impairing their culture expansion.

**Methods:**

Proliferation, differentiation, ROS accumulation, and gene expression were compared between ASC cultures at 35 °C and 37 °C. ASCs isolated either from rat fat depots or from human lipoaspirates were examined in the study.

**Results:**

Rat visceral ASCs (vASCs) cultured at 35 °C demonstrated reduced ROS production and apoptosis and enhanced expansion and adipogenic differentiation compared to vASCs cultured at 37 °C. Similarly, the culture of human ASCs (hASCs) at 35 °C led to reduced ROS accumulation and apoptosis, with no effect on the proliferation rate, compared to hASCs cultured at 37 °C. Comparison of gene expression profiles of 35 °C versus 37 °C vASCs uncovered the development of a pro-inflammatory phenotype in 37 °C vASCs in correlation with culture temperature and ROS overproduction. This correlation was reaffirmed in both hASCs and subcutaneous rat ASCs.

**Conclusions:**

This is the first evidence of the effect of culture temperature on ASC growth and differentiation properties. Reduced temperatures may result in superior ASC cultures with enhanced expansion capacities in vitro and effectiveness in vivo.

## Introduction

Mesenchymal stem cells (MSCs) are multipotent, can be derived from most adult tissues, and have been demonstrated to bear regenerative and immunosuppressive capacities in preclinical models [1]. Although first isolated from the bone marrow, MSCs were also later isolated from adipose tissue and termed adipose-derived stem cells (ASCs) [2, 3]. Clinical utilization of MSCs often requires 1 × 10^6^–5 × 10^6^ cells/kg [4] necessitating significant in vitro expansion of cells prior to their application, increasing the risk of DNA mutation and genetic instability.

Reactive oxygen species (ROS) are a byproduct of mitochondrial oxidative phosphorylation but are also generated as cellular signaling molecules by enzymes such as the family of NOX NADPH oxidases [5]. ROS overproduction leads to various destructive cellular processes, such as aging, DNA damage, and apoptosis [6, 7]. Physiological oxygen levels within the MSC niche were reported to be between 2 and 8% [8]. Elevated oxygen concentrations stimulate increased mitochondrial ROS production by promoting higher ROS leakage from the respiratory chain [9]. Consequently, MSC culture at drastically higher atmospheric oxygen levels (21%), most commonly employed in culture protocols, leads to ROS overproduction, DNA damage, and genetic instability compared to culture under physiological oxygen levels (2–8%) [10–14]. Culture under physiological oxygen conditions also leads to increased proliferation and stem cell potency of both pluripotent [15–17] and adult stem cells [18–24]. Although adaptation of culture conditions to physiological oxygen levels to prevent excess ROS is appealing, reducing oxygen levels from atmospheric levels is demanding and pricy and requires specialized equipment. Thus, simpler and most cost-effective approaches are desirable [25].

Reduced ROS production can theoretically be achieved by decreasing cellular temperature and consequently reducing cellular metabolism and mitochondrial oxygen consumption. Indeed, reduction of body temperature to mild hypothermia was demonstrated to protect against ischemia-induced cardiac damage and stroke [26–28] and to reduce ROS production and NOX activation following stroke [26, 29]. Decreased body temperature was also found to prevent ischemia-induced damage in hibernating animals [30]. This protection results, most probably, from metabolism reduction during hibernation, which leads to reduced mitochondrial activity and reduced ROS production [31, 32].

The effect of low temperature on cellular metabolism is also evident in cultured cells, with various cell types demonstrating lower cellular metabolism in correlation with temperature reduction [33–36]. However, decreased culture temperature also leads to a temperature-dependent reduction in cell proliferation in different cell types [35–39]. For example, the culture of bone marrow-derived MSCs at 32 °C was demonstrated to attenuate ROS accumulation and apoptosis but also cell proliferation [38]. In contrast, long-term bone marrow cultures, first achieved when mesenchymal cells were used to form a niche for hematopoietic stem cells, demonstrated improved culture longevity and hematopoietic cell yields at 33 °C, as compared to the conventional culture conditions of 37 °C [40].

The aim of the current study was to examine whether lowering culture temperature can inhibit the development of oxidative stress and its accompanying phenotype in ASC cultures without hindering their proliferation capacity. We hypothesized that only mildly reducing culture temperature to 35 °C may reduce ROS production in ASCs without hindering their proliferation capacity. Proliferation, differentiation, ROS accumulation, and gene expression were therefore compared between ASC cultures at 35 °C and 37 °C.

## Methods

### Human primary stromal vascular fraction (SVF) cell isolation

Subcutaneous adipose tissue samples were obtained from patients undergoing plastic surgery. All procedures were performed in accordance with the Declaration of Helsinki and approved by the ethics committee of Tel Aviv Sourasky Medical Center. Written, informed consent was obtained from all patients in advance. All samples were waste materials collected as a byproduct of surgery.

### Cell culture of human ASCs

Human primary SVF cells from adipose tissue were maintained in their undifferentiated state in high-glucose Dulbecco’s modified Eagle’s medium (DMEM) (Gibco, Paisley, Scotland, UK), supplemented with 10% fetal calf serum (FCS) (Thermo Scientific HyClone, New Zealand), 60 μg/ml penicillin, 100 μg/ml streptomycin, 50 μg/ml kanamycin, 1 mM sodium pyruvate, 2 mM l-glutamine, and non-essential amino acids, under 10% CO_2_ and atmospheric oxygen conditions at 37 °C. The medium was changed twice a week, and cells were passaged once they reached confluence.

#### Mild hypothermia

Cells were cultured in the medium described above, under atmospheric oxygen, at 35 °C.

### Cell culture of rat ASCs

ASCs were derived from the adipose tissues of Lewis rats (purchased from the Harlan Laboratories Jerusalem, Israel). Cells were isolated from the surgically removed visceral or subcutaneous fat tissue, using 0.1% collagenase (Sigma), and separated from the fat by centrifugation. The cells were cultured in high-glucose DMEM (Gibco), supplemented with 10% FCS (Thermo Scientific HyClone), 60 μg/mL penicillin, 100 μg/mL streptomycin, 50 μg/mL kanamycin, 1 mM sodium pyruvate, 2 mM l-glutamine, and non-essential amino acids, under atmospheric oxygen, at 37 °C. The medium was changed twice a week, and cells were passaged upon reaching confluence.

#### Mild hypothermia

Cells were cultured in the medium described above, under atmospheric oxygen, at 35 °C.

The Tel Aviv Sourasky Medical Center Institutional Animal Care and Use Committee approved all animal experiments, and all experiments were performed in accordance with institutional guidelines.

### Adipogenic differentiation

Confluent cells were cultured in adipogenic medium, composed of DMEM, supplemented with 10% FCS (Thermo Scientific HyClone), 10 μg/mL insulin, 1 × 10^− 6^ M dexamethasone, 0.5 mM 3-isobutyl-1-methyl xanthine (IBMX), and 50 μM indomethacin (all purchased from Sigma). After 21 days, the cells were fixed with 4% formalin (20 min, room temperature [RT]) and stained with 0.5% Oil Red O (10 min, RT) (Sigma). Cells were photographed with an Olympus IX71 microscope (Olympus, Tokyo, Japan) equipped with a DP73 camera. Oil Red O was then extracted with 4% IGEPAL (Sigma) in isopropanol and quantified using a TECAN Infinite M200 plate reader (TECAN, Männedorf, Switzerland, emission 520 nm).

### Osteogenic differentiation

Confluent cells were cultured in StemPro osteogenesis differentiation medium (Gibco). The induction medium was replaced every 3–4 days. After 21 days, the cells were fixed with 4% formalin (20 min at RT) and stained with 2% Alizarin red (Sigma), pH 4.2 (10 min, at RT). Photographs were taken using an Olympus IX71 microscope with a DP73 camera. Alizarin red was extracted with extraction solution (0.5 N HCL and 5% sodium dodecyl sulfate) and quantified at 415 nm using a TECAN Infinite M200 plate reader (TECAN, Männedorf, Switzerland). Reads were normalized according to cell counts.

### Flow cytometry

Apoptosis assessment *by annexin/propidium iodide (PI) staining*: Cells were stained using an annexin-APC/PI detection kit (BioLegend).

Labeled cells were analyzed using a BD FACS Canto II flow cytometer (Becton Dickinson). Data analysis was performed using the FlowJo software (Tree star, Ashland, OR, USA).

### Reactive oxygen species measurements

Cells were trypsinized and incubated (30 min, 37 °C, in the dark) in PBS containing 10 μM 2′,7-dichlorodihydrofluorescein diacetate (H2DCFDA) (Molecular Probes, Carlsbad, CA, USA). H2DCFDA was detected by flow cytometry in a BD FACS Canto II flow cytometer (BD Biosciences), and data were analyzed using the FlowJo software.

### Real-time PCR

RNA was collected from cultured ASCs, during passages 2–5, using a Total RNA Mini Kit (Sigma). cDNA was prepared using M-MLV Reverse Transcriptase (Quanta Biosciences, Gaithersburg, MD, USA), according to the manufacturer’s protocols. Real-time PCR was carried out using the perfeCTa SYBR mix (Quanta Biosciences) and processed using Step One Plus (Applied Biosystems, Foster City, CA, USA), with normalization to Rn18 s. All the primers used in the study are listed in Additional file 1: Table S1.

### CHIP array analysis

Visceral ASCs were grown at 37 °C (triplicates). In addition, visceral ASCs were grown at 35 °C (duplicates). GenEltue Mammalian Total RNA Miniprep kit (Sigma-Aldrich) was used for RNA extraction. Samples were analyzed for gene expression using Affymetrix RaGene-2.1-st-v1 microarray at the Functional Genomics Laboratory, Sackler Faculty of Medicine, Tel Aviv University. Bioinformatics analysis was performed using Partek Genomics Suite (http://www.partek.com/partek-genomics-suite/). In short, cell files were normalized and log2 expression values were obtained, followed by ANOVA statistical evaluation. Differentially expressed genes were extracted using cutoffs *p* value < 0.05 and fold-change difference = 2. A heatmap of differentially expressed genes was generated by Partek Genomics Suite.

All genes that were significantly different between visceral ASCs grown under 35 °C and visceral ASCs grown under 37 °C were analyzed by STRING database [41] protein-protein interaction tool (https://string-db.org/). Enriched processes are shown in Fig. 3.

### Statistical analysis

The statistical significance of the results was determined using a two-tailed Student’s *t* test. *p* values < 0.05 were considered significant.

## Results

### Culture of rat visceral ASCs (vASCs) under 35 °C attenuates oxidative stress, leading to reduced apoptosis and long-term expansion

**Fig. 1:**
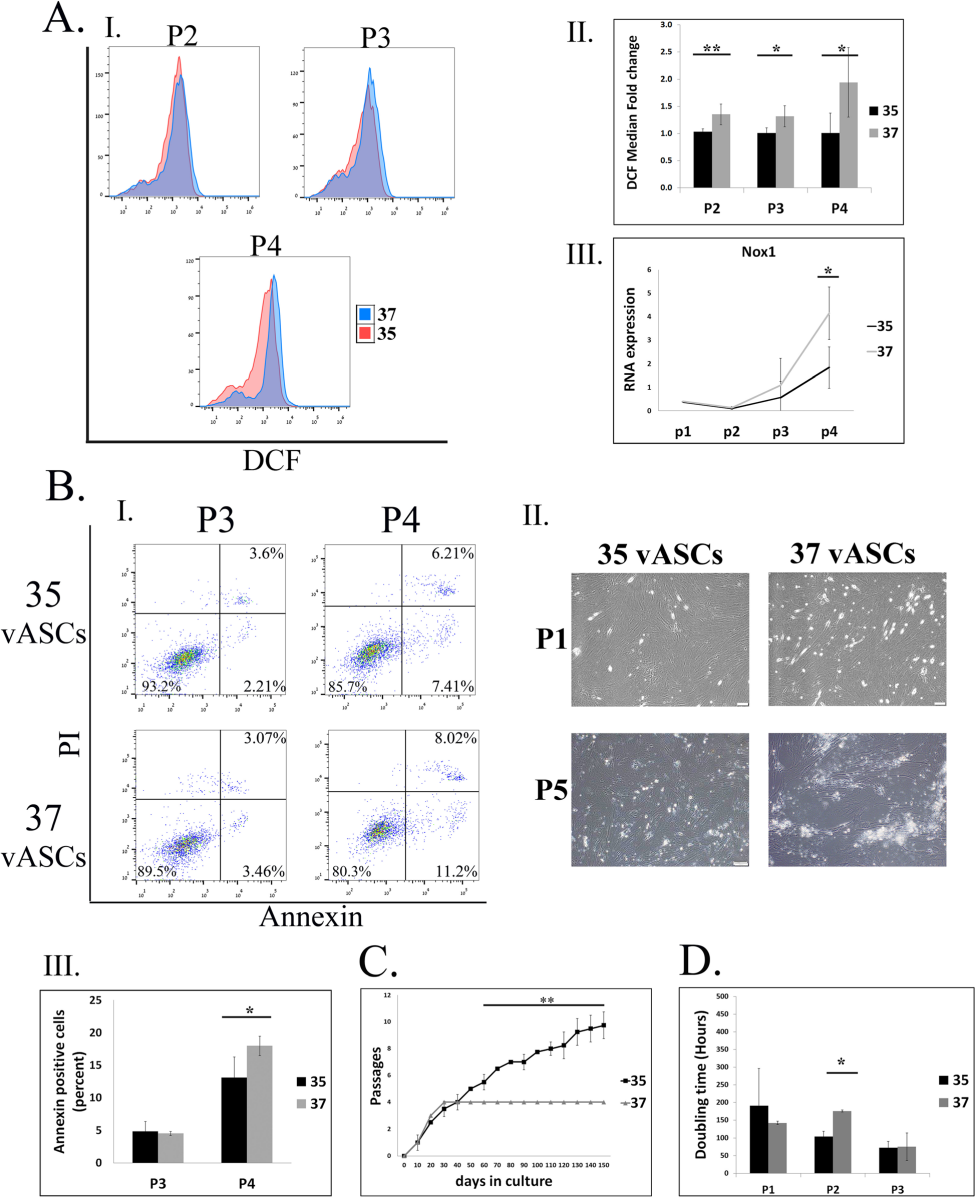
Culture of vASCs at 35 °C attenuates NOX1-dependent oxidative stress and apoptosis, enabling long-term expansion*.* vASCs were propagated at 35 °C or 37 °C. **A** ROS accumulation was detected by DCFDA staining and analyzed by flow cytometry at the indicated passages (P). (AI) Representative FACS analyses. (AII) Summary results of three independent repeats at P2–4. Error bars represent SD. (AIII) qRT-PCR analysis of NOX1 RNA levels in cells grown at 35 °C (black line) versus 37 °C (gray line) at the indicated passages (*n* = two independent repeats). **B** The percentage of apoptotic cells at each passage of cells grown at 37 °C and 35 °C was determined by PI/Annexin staining, followed by FACS analysis. (BI) Representative FACS analyses. (BII) Images of representative 37 °C and 35 °C vASCs at P1 and P5. (BIII) Mean of FACs analyses by-passage rate of apoptosis in 37 °C and 35 °C vASC cultures (*n* = two independent repeats). **C** A growth curve comparing the ability of visceral ASCs to undergo long-term expansion at 37 °C versus 35 °C as evident by the number of passages reached against the time in culture in days (*n* = four independent repeats). Cells were passaged only when reaching confluence. Error bars represent standard deviation (SD). **D** The doubling time of 37OC and 35OC vASC was compared (n=two independent repeats). The doubling time of 37 °C and 35 °C vASC was compared (*n* = two independent repeats). Student’s two-tailed *t* test for equal variance: **p < 0.01; *p < 0.05

Rat visceral ASCs (vASCs) were previously reported by us to repeatedly develop ROS over-accumulation at early passage and apoptosis-induced expansion arrest at passages 4–6 [14]. We therefore chose to examine the effect of reduced culture temperature on ROS production in these cells. In agreement with our previous report [14], vASCs culturing at 37 °C resulted in ROS accumulation in correlation with NOX1 expression (Fig. 1A), a gradual increase in the number of annexin-positive apoptotic cells, reaching approximately 20% at passage 4 (Fig. 1B), and an expansion arrest at passages 4–6 (Fig. 1C). In contrast, vASC culturing at 35 °C, continuously from the time of extraction, was associated with attenuated ROS accumulation in correlation with NOX1 expression, a reduction in apoptosis rates, and long-term expansion of the cell population as compared to vASCs cultured at 37 °C (Fig. 1A, B, and C respectively). Importantly, vASCs culturing at 35 °C did not inhibit their proliferation rate, as demonstrated by a doubling time at passages 1–3 comparable to that of 37 °C vASCs (Fig. 1D). Thus, vASCs cultured at 35 °C display reduced ROS accumulation, which reduces their rate of apoptotic death and enables their long-term expansion.

### Culture under 35 °C enhances vASC fat differentiation potential

**Fig. 2:**
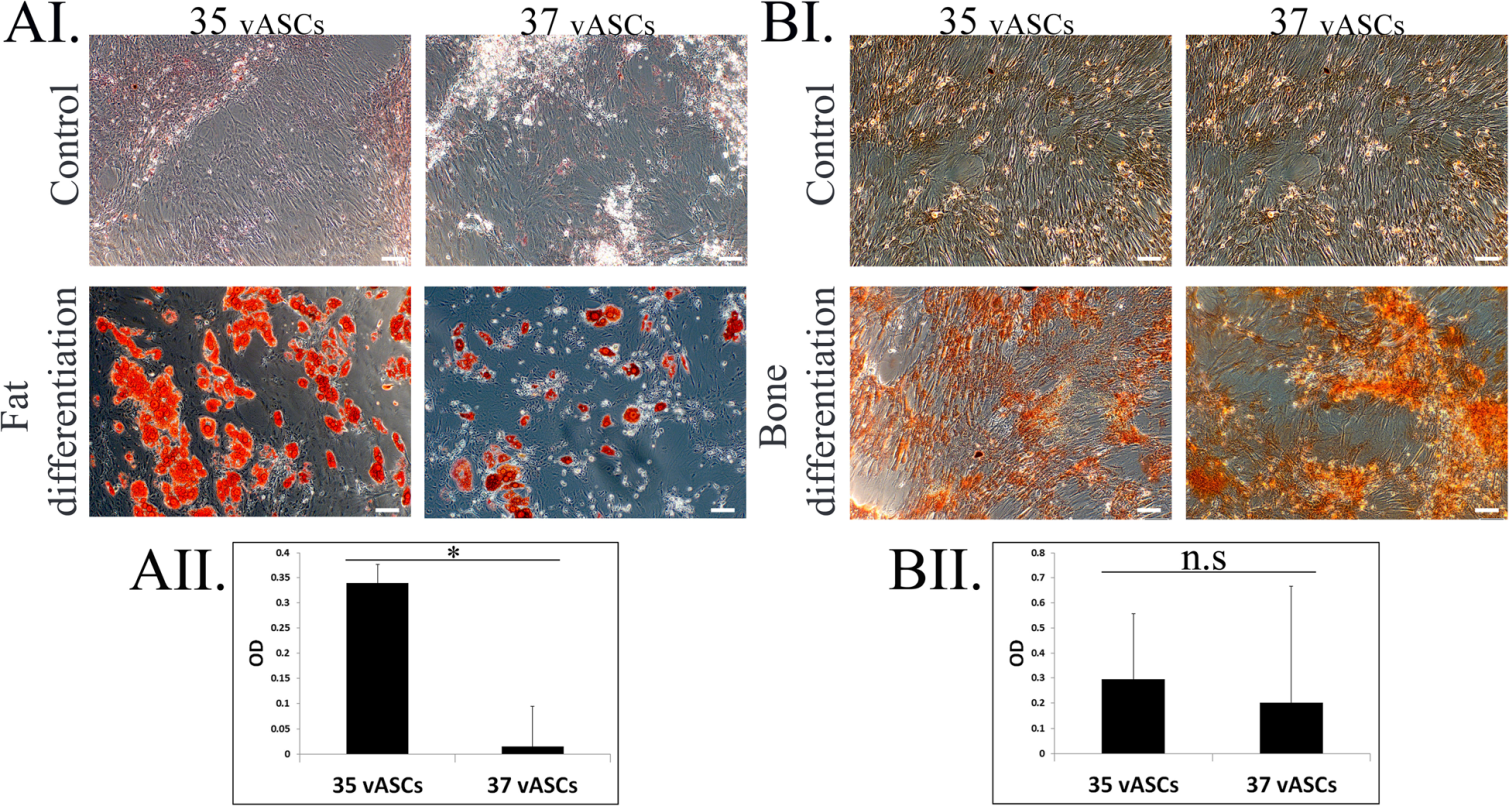
Culture of vASCs at 35 °C significantly improves their fat differentiation potential. The fat (**A**) or bone (**B**) differentiation capacity of vASCs cultured under 35 °C versus 37 °C was determined by culturing cells, at the respective temperatures, for 21 days in fat or bone differentiation mediums respectively. Mean fat and bone differentiation capacity was assessed by staining cells with Oil red O or Alizarin red respectively, photographing the cells (AI and BI) and then extracting and quantifying the stain (AII and BII) (*n* = two independent repeats). *p < 0.05. n.s., difference not statistically significant

We previously described the low fat differentiation potential of vASCs compared to ASCs derived from subcutaneous fat, which correlated with measured levels of NOX1-induced oxidative stress [14]. As can be seen in Fig. 2, expansion of vASCs under 35 °C culture conditions promoted a drastic increase in fat differentiation as compared to vASCs cultured at 37 °C (Fig. 2A) but not of bone differentiation (Fig. 2B).

### Gene array analysis identifies 110 genes significantly up- or downregulated in 37 °C compared to 35 °C vASC cultures

**Fig. 3:**
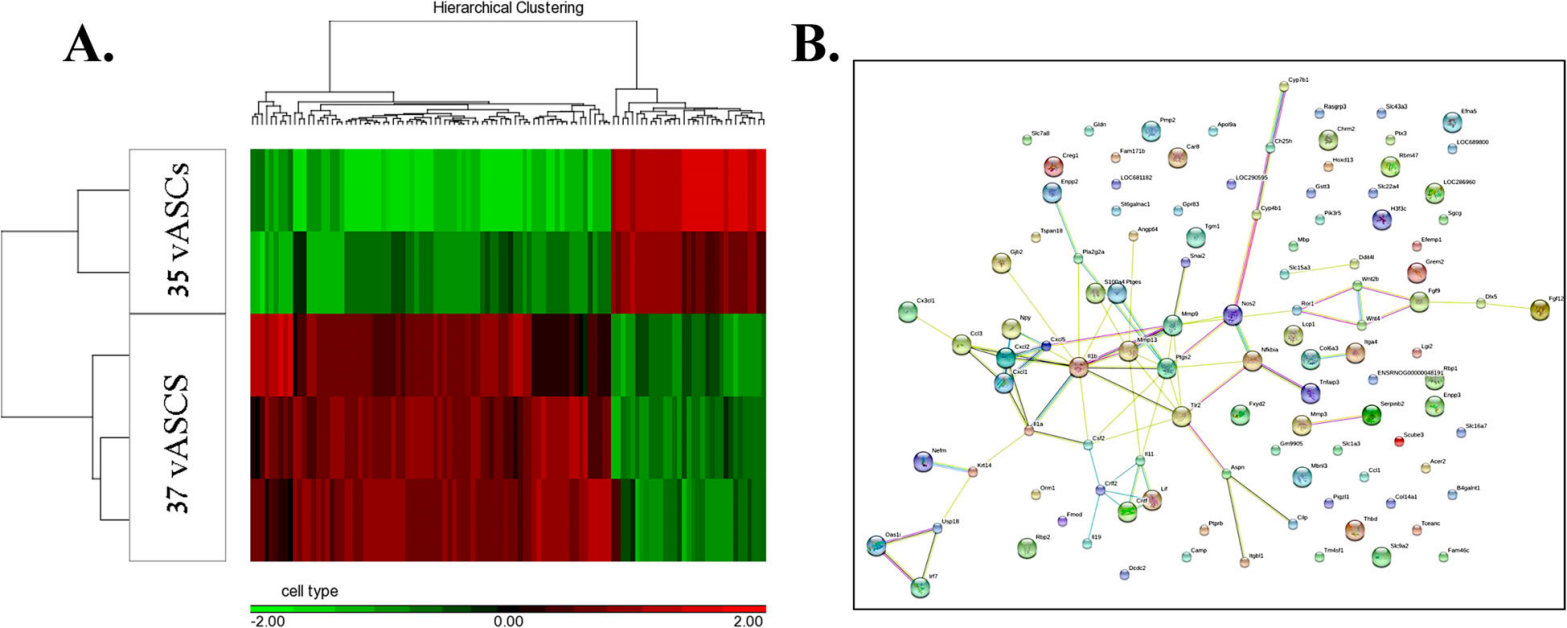
Gene array analysis, protein-protein interactions, and function analysis of vASC differentially expressed genes (35 °C, 37 °C). RNA was produced from passage 3 35 °C and 37 °C vASCs. Samples were analyzed for gene expression using Affymetrix RaGene-2.1-st-v1 microarray. **a** Cluster analysis of the gene array experiment samples showed a clear separation of each treatment (35 °C and 37 °C). Heatmap of 110 differentially expressed genes (*p* < 0.05 and fold-change difference = 2) (**a**). **b** Presentation of the 110 differentially expressed genes (*p* < 0.05 and fold-change difference = 2) responsible for the molecular changes elicited by temperature change (STRING)

In pursuit of the molecular mechanisms responsible for the phenotypic differences between 37 °C vASC and 35 °C vASC cultures, we performed a comparative gene array analysis of vASCs grown under both culture conditions. The gene array analysis was performed on passage 3 cells, which preceded the stage at which 37 °C vASC cultures underwent significant apoptosis and expansion arrest. The analysis categorized the 37 °C vASCs and 35 °C vASCs as two separate populations (Fig. 3a). The gene array analysis identified 110 genes that were significantly (*p* < 0.05) up- or downregulated (> 2-fold change) in 35 °C vASCs as compared to 37 °C vASCs (Fig. 3a).

### Function analysis of differentially expressed genes demonstrates a reduced inflammatory phenotype in 35 °C vASCs

**Fig. 4:**
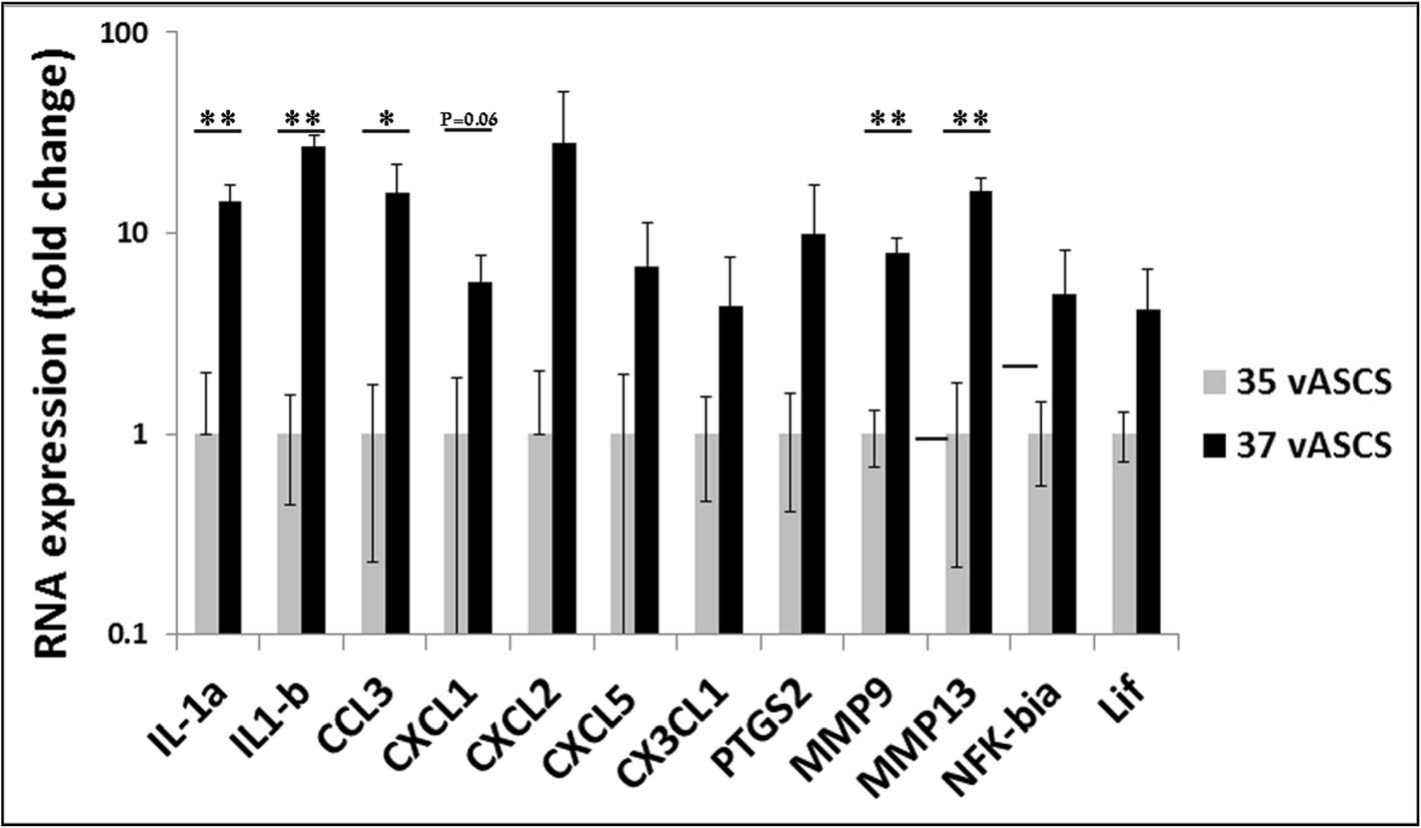
qRT-PCR-based validation of genes highlighted in the functional cluster analysis. RNA was extracted from passage 3 37 °C and 35 °C vASCs, and the gene expression of the indicated genes was compared by qRT-PCR (*n* = 2–3 independent repeats). Gene expression was validated according to the differential expression in the gene array analysis

In order to further characterize the changes in gene expression observed between 35 °C vASCs and 37 °C vASCs, the 110 up/downregulated genes were analyzed using STRING database defining protein-protein interactions and functional enrichment of gene clusters (Fig. 3b). A drastic reduction in the expression level of a range of immune-related factors was noted in 35 °C vASC cultures as compared to 37 °C vASC cultures (Additional file 2: Table S2). An additional gene cluster termed “response to oxygen-containing compounds” containing, amongst other genes, important immune modulators showed reduced gene expression levels in 35 °C vASCs as compared to 37 °C vASCs (Additional file 2: Table S2). Interestingly, temperature reduction was also found to affect cell proliferation, differentiation, and wound healing potential (Additional file 2: Table S2), suggesting a possible influence of culture conditions on the regeneration potential of ASCs. The gene array results (Additional file 3: Table S3) were validated by performing qRT-PCR on 12 representative genes from all four gene groups; the differential gene expression between groups was preserved (Fig. 4).

### ROS accumulation in rat subcutaneous ASCs (scASCs) correlates with increased expression of pro-inflammatory cytokines

**Fig. 5:**
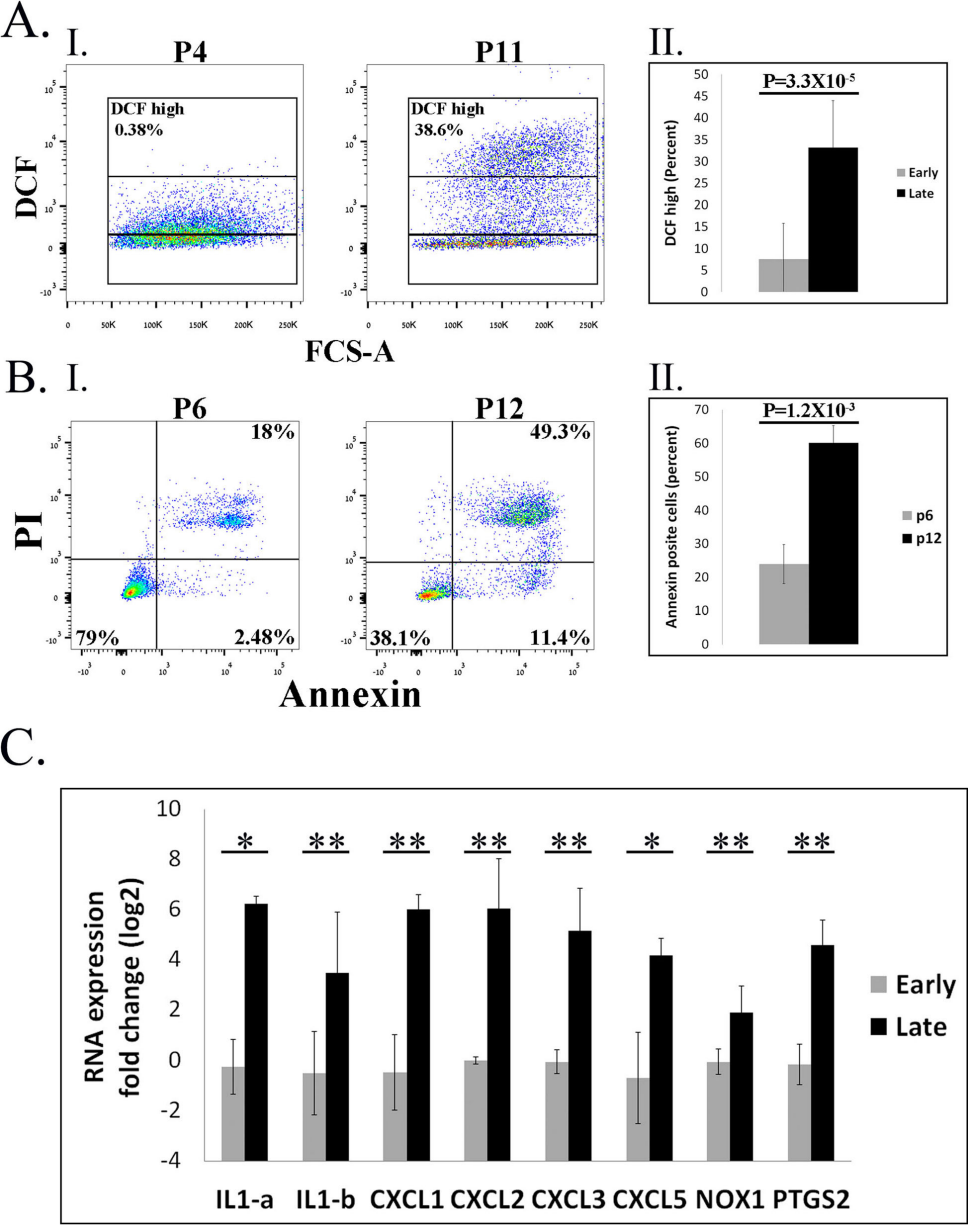
ROS accumulation correlates with the development of a pro-inflammatory phenotype in rat scASCs. Rat scASCs were propagated at 37 °C, and early *p* ≤ 6 and late *p* ≥ 10 cells were harvested and analyzed. **A** ROS accumulation was detected by DCFDA staining and analyzed by flow cytometry at the indicated passages (P). (AI) Representative FACS analyses. (AII) A summary of two independent repeats of the FACS analysis. **B** Comparison of the percentage of apoptotic cells in early versus late passage of cells grown at 37 °C and 35 °C, as determined by PI/Annexin staining, followed by FACS analysis. (BI) Representative FACS analyses. (BII) A summary of two independent repeats. **C** RNA was extracted from early and late passages of 37 °C scASCs, and gene expression of the indicated genes was compared by qRT-PCR (*n* = 2 independent repeats). Data are presented as the mean ± standard deviation. **p < 0.01; *p < 0.05

The results presented thus far demonstrated the tendency of 37 °C vASC cultures to develop ROS over-accumulation/oxidative stress that was correlated with a shift toward a pro-inflammatory phenotype. We previously reported a reduced tendency of scASCs to accumulate ROS at passages 1–6 compared to vASCs [14]. However, As can be seen in Fig. 5A, rat scASCs cultured at normal culture conditions (37 °C) demonstrated increased ROS accumulation (Fig. 5A) and apoptosis (Fig. 5B) at late passages (> 10) compared to their early counterparts (passages 4–6). Thus, to verify the correlation between ROS accumulation and the development of a pro-inflammatory phenotype, we compared the pro-inflammatory phenotype of early versus late scASCs. As seen with vASCs, increased ROS accumulation at late scASC passages was associated with an increased pro-inflammatory phenotype (Fig. 5C).

### Culture of human subcutaneous ASCs at 35 °C attenuates oxidative stress development, leading to reduced apoptosis without impairing expansion

**Fig. 6:**
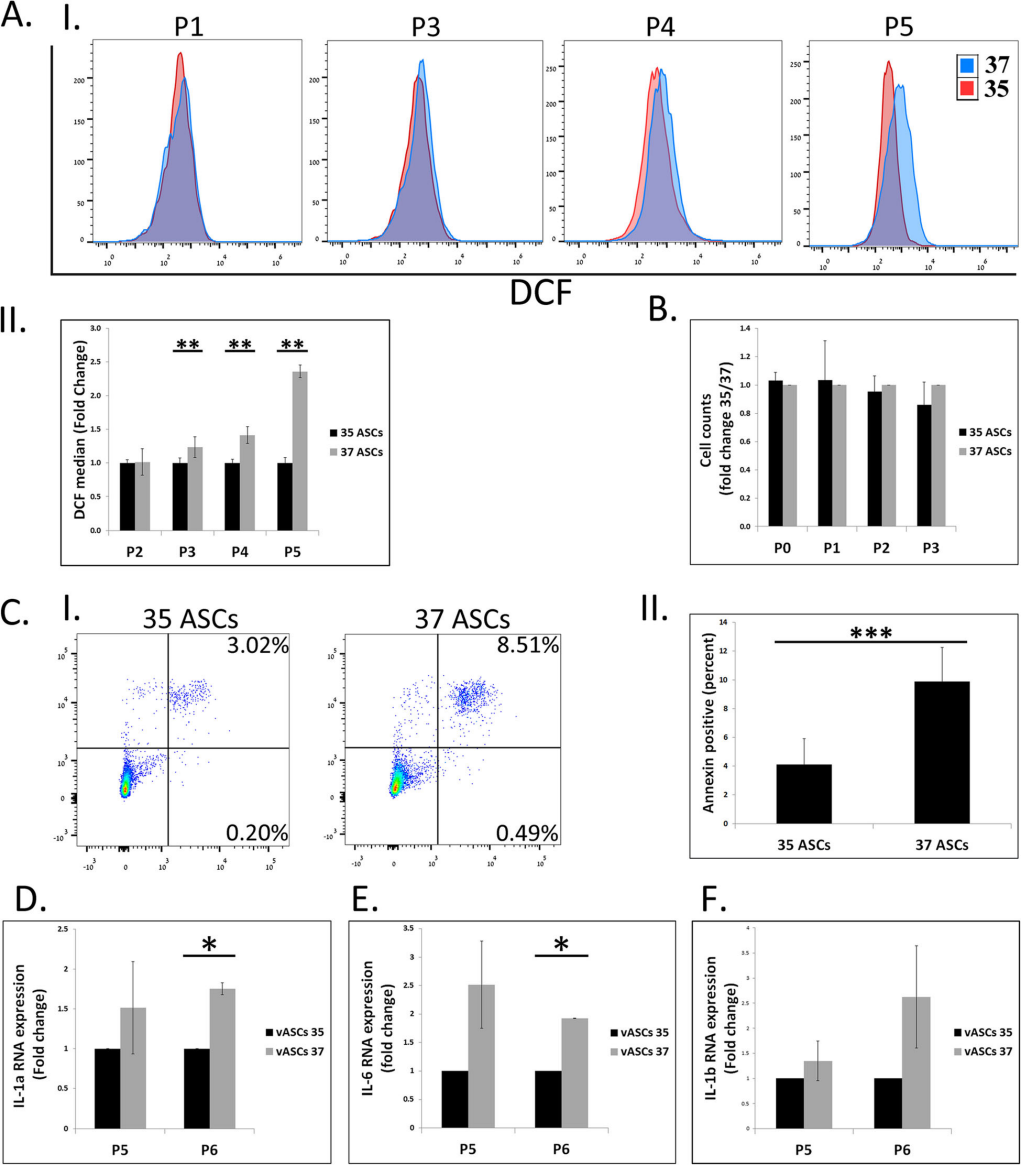
Culture of human subcutaneous ASCs at 35 °C attenuates ROS accumulation and apoptosis without affecting cell expansion. Human ASCs were isolated from lipoaspirates, seeded, and immediately cultured either at a normal culture temperature (37 °C) or at a mildly reduced temperature (35 °C). **A** Comparison of ROS accumulation between ASCs isolated from the same lipoaspirate and grown at either 37 °C or 35 °C was performed by DCFDA staining and its analysis by flow cytometry, at the indicated passages (P). (AI) Representative FACS analyses. (AII) Summary of three independent repeats from three different patients, at P2–4, and one repeat at P5. Data are presented as the mean ± standard deviation. **B** Cells were counted at each passage and reseeded at equal numbers. Cell counts at each passage are presented (*n* = 4 independent repeats). **c** Comparison of the percentage of apoptotic ASCs at each passage of cells grown at 37 °C and 35 °C was performed by PI/Annexin staining, followed by FACS analysis. (CI) A representative FACS analysis. (CII) Summary results of three biological repeats from one patient at P5. Similar differences in the level of apoptosis between 37 and 35 °C ASC cultures were obtained from three independent patients but at different passages. **D**–**F** RNA was extracted at different passages and the level of expression of the indicated genes was compared between 37 and 35 °C ASCs (*n* = 2). Data are presented as the mean ± standard deviation. ***p < 0.001; **p < 0.01; *p < 0.05

Clinically relevant human ASCs are generally produced from subcutaneous fat extracted by different liposuction techniques. Therefore, we decided to examine whether our findings in rat vASCs and scASCs also apply to human subcutaneous ASCs. To this end, we examined the effect of reduced culture temperature on the phenotype of subcutaneous ASCs by comparing their long-term expansion at 37 °C and 35 °C. Increased ROS accumulation in 37 °C compared to 35 °C ASCs was already evident at passage 3 and was further enhanced with increasing passages (Fig. 6A). Importantly, however, the culture of ASCs at 35 °C did not affect their expansion rate, as evident from cell quantities comparable to those of 37 °C ASCs at passages 0–3 (Fig. 6B). At passage 5, ROS over-accumulation in 37 °C ASCs led also to their increased apoptosis as compared to 35 °C ASCs (Fig. 6C). The correlation between ROS accumulation and the development of a pro-inflammatory phenotype was also demonstrated, with human ASCs cultured at 37 °C exhibiting increased expression of pro-inflammatory cytokines (IL-1b, IL-1a, and IL-6) as compared to ASCs cultured at 35 °C (Fig. 6D–F).

## Discussion

We and others have previously described the tendency of ASCs to accumulate ROS during long-term culturing, leading to DNA damage, apoptosis, expansion arrest, and reduced fat differentiation potential [12, 14]. In the current study, we showed, for the first time, that a 2 °C reduction in the culture temperature inhibits ROS over-accumulation in both human subcutaneous ASCs and rat vASCs, resulting in reduced apoptosis and improved long-term expansion. In addition, the culture of rat vASCs at 35 °C led also to their enhanced fat differentiation compared to 37 °C vASCs. Gene array analysis demonstrated reduced expression of many immune modulators in 35 °C versus 37 °C ASC cultures, indicating a reduced inflammatory phenotype under 35 °C culture conditions. Functional analysis further identified reduced expression of a cluster of “response to oxygen-containing compounds” genes in 35 °C vASCs compared to 37 °C vASCs, correlating the increased inflammatory phenotype in 37 °C vASCs with their increased ROS production and oxidative stress. The correlation between ROS overproduction and the pro-inflammatory phenotype was further corroborated by the observed increased expression of pro-inflammatory genes in both human and rat subcutaneous ASCs in response to ROS accumulation. Thus, reducing culture temperature can enhance ASC quality and quantity and prevent culture-induced oxidative stress and cell damage, which may all impact their clinical effectiveness.

Elevated oxygen concentrations are known to stimulate increased mitochondrial ROS production by promoting higher ROS leakage from the respiratory chain [9]. In turn, the increased oxygen levels during the transition of MSC progenitor cells from their physiological niche conditions (2–8% oxygen) to culture conditions (atmospheric oxygen 21%) trigger enhanced mitochondrial ROS production and ROS over-accumulation [10–14]. One viable approach to attenuate mitochondrial ROS accumulation during MSC culture is reducing culture temperature, which, in turn, will decrease cellular metabolism as was reported for various cell types [33–36]. Reduced ROS accumulation in 32 °C bone marrow-derived MSC cultures was previously reported [38]. Yet, the culture of both bone marrow-derived MSCs and additional cell types at temperatures below 34 °C was also reported to inhibit cell proliferation, [35, 38] hindering cell expansion. In contrast, culture at temperatures between 34 and 35.5 °C was reported to promote similar cell proliferation rates as cultures at 37 °C, despite a significant reduction in cellular oxygen and glucose consumption [37, 39]. We therefore chose to assess long-term expansion of ASCs under mild hypothermia (35 °C). These culture conditions were able to salvage both rat and human ASCs from ROS overproduction and consequential apoptosis. In rat vASCs, reducing culture temperature from 37 to 35 °C also prevented apoptosis-induced expansion arrest that was previously observed in 37 °C vASCs, enabling long-term expansion, without affecting proliferation rates; the 35 °C vASC cultures proliferated just as effectively as passage 0–3 37 °C vASC cultures prior to expansion arrest. Similar to vASCs, no reduction in the proliferation of 35 °C human ASCs was observed compared to 37 °C human ASCs.

We previously reported that ROS overproduction in vASCs was accompanied by increased expression of NOX1 but not of NOX2 or NOX4. NOX family members are an important source of intracellular ROS pointing to NOX1 involvement in ROS accumulation [14]. This was verified when vASCs that were grown under 3% oxygen conditions and expanded long term displayed reduced NOX1 expression and decreased ROS accumulation [14]. NOX1 involvement in vASC cytostasis was reaffirmed when cells that were expanded under normal oxygen conditions (21%) in the presence of a specific NOX1 inhibitor, ML171, demonstrated reduced ROS accumulation, reduced apoptosis, and long-term expansion [14]. In the current study, we demonstrated that vASC culturing at 35 °C was associated with an attenuated ROS accumulation in correlation with NOX1 expression, a reduction in apoptosis rates, and long-term expansion of the cell population as compared to vASCs cultured at 37 °C. This indicates that ROS decreased accumulation in 35 °C vASCs compared to 37 °C vASCs resulted, at least in part, from the reduction of NOX1 expression under 35 °C culture conditions.

We previously showed a direct correlation between the reduction of ROS levels and fat differentiation potential in 37 °C vASC cultures upon administration of a NOX1-specific inhibitor [14]. Enhanced fat differentiation potential in correlation with reduced ROS accumulation was similarly observed in scASCs [14]. In the current study, reduced culture temperature increased vASC fat differentiation capabilities in correlation with reduced ROS accumulation. It is thus reasonable to assume that the markedly improved fat differentiation potential of 35 °C vASC cultures was a direct result of reduced ROS production. It has been generally agreed that ROS accumulation positively regulates fat differentiation [42], although the exact source of ROS during adipogenesis is still controversial. Thus, unlike the temporal physiological increase in ROS levels, previously reported during fat differentiation, the increased chronic and toxic accumulation of ROS in 37 °C vASC cultures seems to disrupt many physiological processes, including fat differentiation processes.

Comparison of the gene expression pattern of 37 °C versus 35 °C ASCs revealed an increased expression of various positive modulators of immune processes, including the pivotal IL-1a and IL-1b cytokines and various chemokines in 37 °C ASCs. Thus, culturing ASCs under slightly hypothermic conditions reduces their inflammatory phenotype as compared to 37 °C ASCs. In recent years, MSCs have gained much attention due to their potential application as immunomodulators for the treatment of immune-mediated diseases such as autoimmune diseases, graft-versus-host disease (GvHD), and inflammatory diseases [43]. In such cases, it would seem that the reduced inflammatory phenotype of 35 °C vASCs would provide a clinical advantage.

In an earlier study [14], we showed that early-passage scASCs cultured at 37 °C generated less ROS as compared to 37 °C vASCs and consequently demonstrated reduced apoptosis and enhanced expansion and fat differentiation than the former [14]. In the current study, similar to scASCs, vASCs cultured at 35 °C demonstrated reduced ROS accumulation that correlated with their reduced apoptosis and enhanced expansion and fat differentiation compared to 37 °C vASCs. Thus, we speculate that most of the changes in gene expression levels measured in 35 °C vASCs occurred in response to reduced ROS production in these cells. Accordingly, analysis of the gene array results also uncovered enhanced expression of genes associated with response to oxygen, the majority of which were immune modulators or genes involved in immune regulation, in 37 °C vASCs. Therefore, since 35 °C culture conditions also led to a significant reduction in ROS accumulation in vASCs, we wanted to determine whether ROS accumulation played a direct role in the development of the pro-inflammatory phenotype of 37 °C vASCs. To this end, we compared the expression of pro-inflammatory immune modulators between early- and late-passage (> 10) rat scASCs cultured at 37 °C, which display increased oxidative stress at later passages. Indeed, a significant increase in the pro-inflammatory phenotype was found in late- compared to early-passage scASCs, in correlation with oxidative stress enhancement, indicating that ROS accumulation and not culture temperature was responsible for the phenotypic shift. In accordance, 35 °C human ASCs cultures demonstrated significantly reduced ROS production in correlation with the reduced expression of the pro-inflammatory cytokines IL-1a, IL-1b, and IL-6 compared to 37 °C human ASCs. In conclusion, we propose that the development of intracellular oxidative stress in ASC cultures elicits increased expression of various immune modulators, with a tendency toward a pro-inflammatory phenotype.

## Conclusions

Thus, a mild temperature shift in ASC cultures can induce profound effects on their in vitro phenotype without affecting their proliferation rate, possibly affecting the cells’ in vivo clinical efficacy. Given their highly plastic nature, the culture conditions of MSCs, in general, and of ASCs, in particular, must be strictly controlled to avoid inter-batch variability. In addition, given the undisputed harmful effect of oxidative stress on cells, we speculate that a mildly reduced culture temperature may serve as a simple means of preventing ROS overproduction in ASCs.

## Supplementary information

**Additional file 1: Table S1.** Primer list.

**Additional file 2: Table S2.** Function analysis of the differentially expressed genes between 37 °C and 35 °C ASCs.

**Additional file 3: Table S3.** Gene array results of the main pro-inflammatory genes appearing in the function analysis of the differentially expressed genes between 37 °C and 35 °C ASCs.

## Data Availability

The datasets used and/or analyzed during the current study are available from the corresponding author on reasonable request.

## Abbreviations

ASCs: Adipose-derived stem cells
DMEM: Dulbecco’s modified Eagle’s medium
FCS: Fetal calf serum
GvHD: Graft-versus-host disease
H2DCFDA: 2′,7-Dichlorodihydrofluorescein diacetate
MSCs: Mesenchymal stem cells
PI: Propidium iodide
ROS: Reactive oxygen species
RT: Room temperature
scASCs: Subcutaneous ASCs
SVF: Stromal vascular fraction
vASCs: Rat visceral ASCs
