# Supplementary material for: Reduced culture temperature attenuates oxidative stress and inflammatory response facilitating expansion and differentiation of adipose-derived stem cells
Source: Stem Cell Res Ther. 2020 Jan 23;11:35. doi: 10.1186/s13287-019-1542-0 (PMC6979291; doi:10.1186/s13287-019-1542-0)
Supplement: Supplementary file 1 — Additional file 1: Table S1. Primer list. [file 13287_2019_1542_MOESM1_ESM.pdf]

Gene	Primers
Nox1 F	CGGGGTCAAACAGAAGAGAG
Nox1 R	TGAGGACTCCTGCAACTCCT
CXCL2 F	TGGTTCAGAGGATCGTCCA
CXCL2 R	TCTTTGATTCTGCCCGTTG
IL-Iβ F	CAGGAAGGCAGTGTCACTCA
IL-Iβ R	AAAGAAGGTGCTTGGGTCCT
IL-1α F	GAGATTCCGGAAACACCAAA
IL-1α R	GAAAGCTGCGGATGTGAAGT
CXCL1 F	GCACCCAAACCGAAGTCATA
CXCL1 R	GGGGACACCCTTTAGCATCT
CX3CL1 (Fractalkine) F	ATCTGTGTACTCTGCTGGCG
CX3CL1 (Fractalkine) R	AGCAAGGTCACTGGGATTGG
CCL3 F	CTTCTCCTATGGACGGCAAA
CCL3 R	CGGTTTCTCTTGGTCAGGAA
CXCL5 F	GTTCACACTGCCACAGCATC
CXCL5 R	GCGATCATTTTGGGGTTAAT
CXCL2 F	TGGTTCAGAGGATCGTCCA
CXCL2 R	TCTTTGATTCTGCCCGTTG
MMP9 F	CAATCCTTGCAATGTGGATG
MMP9 R	TTCCTCCGTGATTCGAGAAC
MMP13 F	AAAGACTATCCCCGCCTCAT
MMP13 R	TGGGCCCATTGAAAAAGTAG
PTGS2 F	TTCCAAACCAGCAGGCTCAT
PTGS2 R	AAAAGCAGCTCTGGGTCGAA
NFK-bia F	GTACCCGGATACAGCAGCAG
NFK-bia R	AGGGCAACTCATCTTCCGTG
Lif rattus F	GTGCCAATGCCCTCTTTATTTC
Lif R	GCATGGAAAGGTGGGAAATC
Rn18s F	CGAAAGCATTTGCCAAGAAT
Rn18s R	AGTCGGCATCGTTTATGGTC
