# Supplementary material for: Reduced culture temperature attenuates oxidative stress and inflammatory response facilitating expansion and differentiation of adipose-derived stem cells
Source: Stem Cell Res Ther. 2020 Jan 23;11:35. doi: 10.1186/s13287-019-1542-0 (PMC6979291; doi:10.1186/s13287-019-1542-0)
Supplement: Supplementary file 2 — Additional file 2: Table S2. Function analysis of the differentially expressed genes between 37 °C and 35 °C ASCs. [file 13287_2019_1542_MOESM2_ESM.pdf]

	Pathway description	Observed gene count	False discovery rate	Matching proteins in your network (labels)
**Group I: Immune response**	positive regulation of leukocyte migration	8	5.71E-07	Ccl3,Cx3cl1,Cxcl1,Cxcl2,Cxcl5,Il1a,Il1b,Mmp9
	positive regulation of immune system process	10	3.62E-05	Ccl3,Csf2,Cx3cl1,Cxcl1,Cxcl2,Cxcl6,Il1a,Il1b,Mmp9, Nfkbia
**Group II: Oxygen-related**	response to oxygen-containing compound	16	0.00118	Ccl3,Csf2,Cxcl1,Cxcl2,Cxcl5,Cyp7b1,Gjb2,Il1a,Il1b,Mbp, Mmp9,Nefm,Nfkbia,Orm1,Ptgs2,Wnt4
	response to hypoxia	7	0.0172	Angptl4,Cx3cl1,Il1a,Il1b,Mmp13,Mmp9,Ptgs2
**Group III: Stress**	response to heat	5	0.00335	Cxcl1,Cxcl2,Il1a,Il1b,Mmp9
	response to stress	17	0.0148	Angptl4,Ccl3,Cx3cl1,Cxcl1,Cxcl2,Cxcl5,Fgf12,Fmod,Gjb2 ,Il1a,Il1b,Mmp13,Mmp9,Nefm,Orm1,Serpinb2,Wnt4
**Group IV: Regenerative /wound healing**	regulation of cell proliferation	13	0.000555	Cntf,Csf2,Cx3cl1,Cxcl1,Cxcl2,Cxcl5,Fxyd2,Il1b,Mmp9, Nfkbia,Pla2g2a,Ptgs2,Snai2
	regulation of cell differentiation	16	0.0000169	Ccl3,Cntf,Cx3cl1,Cxcl5,Dlx5,Efemp1,Enpp3,Fgf9,Il1b,Lif, Mbp,Nfkbia,Pla2g2a,Ptgs2,Rbp1,Snai2
	wound healing	6	0.0177	Cx3cl1,Cxcl2,Fmod,Il1a,Il1b,Serpinb2
