# Supplementary material for: Reduced culture temperature attenuates oxidative stress and inflammatory response facilitating expansion and differentiation of adipose-derived stem cells
Source: Stem Cell Res Ther. 2020 Jan 23;11:35. doi: 10.1186/s13287-019-1542-0 (PMC6979291; doi:10.1186/s13287-019-1542-0)
Supplement: Supplementary file 3 — Additional file 3: Table S3. Gene array results of the main pro-inflammatory genes appearing in the function analysis of the differentially expressed genes between 37 °C and 35 °C ASCs. [file 13287_2019_1542_MOESM3_ESM.pdf]

	GENES	Fold change vASCs 37/35	pv
1	IL-1a	7.80	0.0008
2	IL1-b	15.87	0.0002
3	CCL3	6.14	0.0252
4	CXCL1	3.63	0.0255
5	CXCL2	11.46	0.0028
6	CXCL5	6.05	0.0137
7	CX3CL1	2.42	0.0146
8	PTGS2	3.67	0.0037
9	MMP9	3.34	0.0112
10	MMP13	4.60	0.0029
11	NFKbia	2.50	0.0002
12	Lif	2.80	0.0009
